# Prognostic value of low skeletal muscle mass in patient treated by exclusive curative radiochemotherapy for a NSCLC

**DOI:** 10.1038/s41598-021-90187-6

**Published:** 2021-05-20

**Authors:** R. Mallet, P. Decazes, R. Modzelewski, J. Lequesne, P. Vera, B. Dubray, S. Thureau

**Affiliations:** 1 Department of Radiation Oncology, Centre Henri Becquerel and Rouen University Hospital, & QuantIF–LITIS [EA (Equipe d’Accueil) 4108], Rouen, France; 2 Department of Nuclear Medicine, Centre Henri Becquerel and Rouen University Hospital, & QuantIF–LITIS [EA (Equipe d’Accueil) 4108–FR CNRS 3638], Faculty of Medicine, University of Rouen, Rouen, France; 3 Clinical Research Department, Centre Henri Becquerel, Rouen, France

**Keywords:** Non-small-cell lung cancer, Radiotherapy

## Abstract

Low skeletal muscle mass is a well-known prognostic factor for patients treated for a non-small-cell lung cancer by surgery or chemotherapy. However, its impact in patients treated by exclusive radiochemotherapy has never been explored. Our study tries to evaluate the prognostic value of low skeletal muscle mass and other antropometric parameters on this population. Clinical, nutritional and anthropometric date were collected for 93 patients treated by radiochemotherapy for a NSCLC. Anthropometric parameters were measured on the PET/CT by two methods. The first method was a manual segmentation at level L3, used to define Muscle Body Area (MBA_L3_), Visceral Fat Area (VFA_L3_) and Subcutaneous Fat Area (SCFA_L3_). The second method was an software (Anthropometer3D), allowing an automatic multislice measurement of Lean Body Mass (LBM_Anthro3D_), Fat Body Mass (FBM_Anthro3D_), Muscle Body Mass (MBM_Anthro3D_), Visceral Fat Mass (VFM_Anthro3D_), and Sub-Cutaneous Fat Mass (SCFM_Anthro3D_) on the PET/CT. All anthropometrics parameters were normalised by the patient's height. The primary end point was overall survival time. Univariate and then stepwise multivariate cox analysis were performed for significant parameters. Finally, Spearman's correlation between MBA_L3_ and MBM_Anthro3D_ was assessed. Forty-one (44%) patients had low skeletal muscle mass. The median overall survival was 18 months for low skeletal muscle mass patients versus 36 months for non-low skeletal muscle mass patients (*p* = 0.019). Low skeletal muscle mass (HR = 1.806, IC95% [1.09–2.98]), serums albumin level < 35 g/l (HR = 2.203 [1.19–4.09]), Buzby Index < 97.5 (HR = 2.31 [1.23–4.33]), WHO score = 0 (HR = 0.59 [0.31–0.86] and MBM_Anthro3D_ < 8.56 kg/m^2^ (HR = 2.36 [1.41–3.90]) were the only significant features in univariates analysis. In the stepwise multivariate Cox analysis, only MBM_Anthro3D_ < 8.56 kg/m^2^ (HR = 2.16, *p* = 0.003) and WHO score = 0 (HR = 0.59, *p* = 0.04) were significant. Finally, muscle quantified by MBA_L3_ and MBM_Anthro3D_ were found to be highly correlated (Spearman = 0.9). Low skeletal muscle mass, assessed on the pre-treatment PET/CT is a powerful prognostic factor in patient treated by radiochemotherapy for a NSCLC. The automatic software Anthropometer3D can easily identify patients a risk that could benefit an adapted therapy.

## Introduction

Lung cancer is one of the most common cancer in USA in 2018, and the deadliest cancer^1^. Despite progress in the care of this pathology, the prognostic remains poor with a 5-Years overall survival rate of 18.6%^2^.

For patients with a disease who can benefit from the surgery because of a locally unresectable lesion or with a contraindication for surgery, the standard of care is radiotherapy, with or without platin-based chemotherapy^3^ (CRT). This treatment can be given sequentially or concurrently, usually at a dose of 60–66 Gy in 30–33 fractions over 6–7 weeks. A radiotherapy boost is not recommended, a trial led to poorer survival^4^. Pretherapeutic evaluation includes cardiac, pulmonary function assessments and general status review. Nutritional status is a well-known prognostic factor in cancer, with an influence on toxicities, response to treatment, surgical complications and overall survival in several localisations^5,6^. The usual tools are Body Mass Index, weight history and albumin serum level. But in the last decades, there have been an increased interest for qualitative nutritional assessment, and particularly for skeletal muscle mass and sarcopenia.

Sarcopenia is a multifactorial metabolic syndrome characterized by a loss of skeletal muscle mass with or without loss of fat mass, associated with reduced physical function and poor tolerance to anticancer therapy^8–12^. Prado et al. showed that sarcopenia at baseline, defined by a quantitative approach, assessed by CT-scan of Skeletal Muscle Area (SMA) on the third lumbar vertebra, is a powerful prognostic factor for solid tumours of the respiratory and gastrointestinal tracts^10^. For lung cancer, prevalence of sarcopenia at diagnosis varies between 24 and 74%^11,12^. To our knowledge, the prognostic impact of sarcopenia (defined by Prado as low skeletal muscle mass) on overall survival in patient treated by exclusive radiochemotherapy, and more generally anthropometrical parameters evaluated by CT-scan^10^, has not yet been evaluated.

The primary objective was to evaluate the impact of low muscular mass (by Prado and Baumgartner) with two segmentation methods, in a population of patients treated by exclusive curative radio-chemotherapy for localised NSCLC.

The second objectives were to evaluate the impact of clinical, nutritional and anthropometric parameters (evaluate with the same segmentation methods than muscular mass).

## Methods

### Patients and procedures

A single-center retrospective open study was performed in the Henri Becquerel Cancer Center, Rouen, France. A computerized review of patients treated for a NSCLC was undertaken. The population included all new patients referred to the radiotherapy department with a NSCLC between 2012 and 2015. The inclusion criteria were histologically confirmed NSCLC cancer, pre-therapeutic extension assessment by ^18^FDG-PET/CT with available images, and chemoradiotherapy for a curative aim treatment exclusively. Exclusion criteria included surgery, palliative treatment and presence of metastasis. Clinical, morphological, nutritional, and imaging parameters at baseline were collected.

As regarding baseline clinical data, age, sex, WHO performance status, histological subtypes, cancer location and TNM stage were collected. The baseline nutritional parameters were weight, weight loss history (over the last 6 months), size, body mass index (BMI), serum albumin levels, and Nutritional Risk Index^13^.

We also collected treatment parameters: chemotherapy protocol, number of chemotherapy lines, type of treatment (sequential/concurrent CRT, exclusive radiotherapy) radiotherapy dose (Gy), number of treatment days, volume treated at 60–66 Gy (cm^3^). Using Common Terminology Criteria for Adverse Events (CTCAE) V4.03, we collected grade ≥ 3 toxicities of the treatment for cardiac, gastric, pneumologic, biological and nephrologic events (and their impact on CRT: radiotherapy interruption for more than 3 days, chemotherapy interruption, reports or dose reduction of the chemotherapy).

With ^18^FDG-PET/CT, we used CT-slice to assess skeletal muscle mass by two methods, described further, and to measure Standardized Uptake Value Maximum (SUV_Max_).

From our institution follow, we collected time to progression (define on diagnose provide by ^18^FDG-PET/CT), and overall survival.

### Anthropometric parameters

Skeletal muscle mass was assessed using the ^18^FDG-PET/CT and analysed by homemade plugin running on the Telemis 4.7-Picture Archiving and Communication System (PACS). Skeletal muscle was identified and quantified by use of Hounsfield Unit (HU) thresholds (− 29 to + 150)^14^. The L3 skeletal muscles were psoas, paraspinal muscles and abdominal wall muscles. We segmented the L3 skeletal muscle cross-sectional area on two adjacent cross-sectional images of the third lumbar vertebra, and the mean value for both images was defined as skeletal muscle lumbar 3 areas (cm^2^). This value was normalized for stature to estimate the Skeletal Muscle Index (SMI, cm^2^/m^2^)^14^. Using the sex specific cut-offs defined by Prado et al., low skeletal muscle mass was defined as an SMI < 52.4cm^2^/m^2^ for men and < 38.5 cm^2^/m^2^ for women^10^. Using the same method, we measured Visceral Fat Mass (VFM) and Subcutaneous Fat Mass (SCFM).

**Figure 1:**
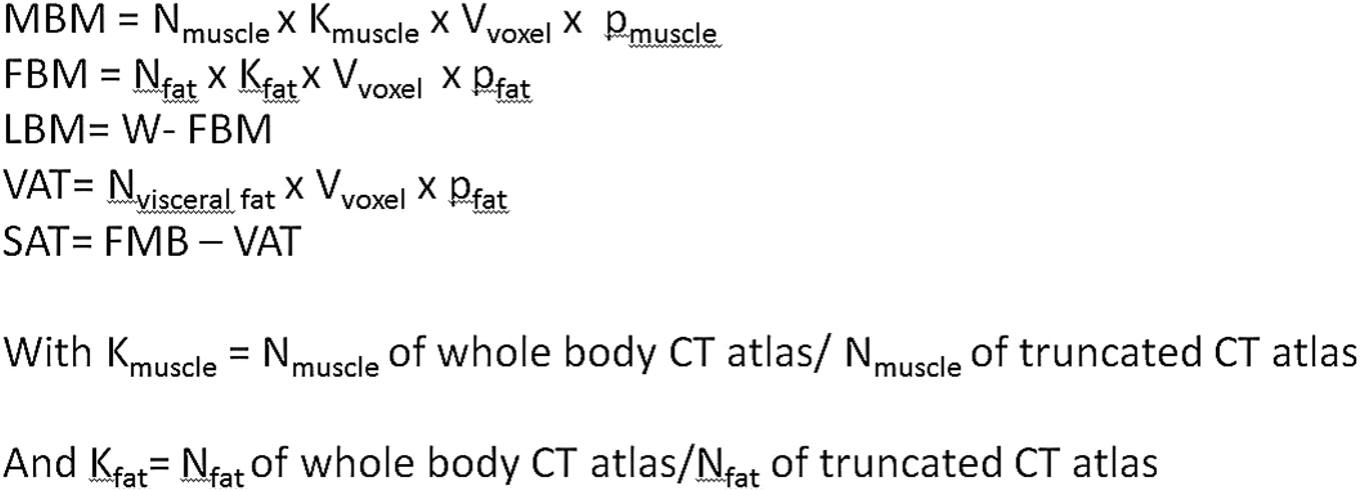
Calcul of anthropometric parameters with Anthropometer3D. Vvoxel is the volume of one voxel (ml), ρ is the density of the tissue (1.06 g/ml for muscle, 0.923 g/ml for fat, and N is the number of voxel for each tissue.

We also measured anthropometric parameters with a second method, using Anthropometer3D software developed by our team, which allows a multi-slice measurement of several anthropometrics parameters on the CT of ^18^FDG-PET/CT. This software uses a multi-atlas segmentation method combined with an extrapolation of the body parts outside the field of acquisition. Lean Body Mass (LBM), Fat Body Mass (FBM), Muscle Body Mass (MBM), Visceral Fat Mass (VFM) and Subcutaneous Fat Mass (SCFM) can be measured automatically by this software. ^18^FDG-PET/CT was acquired from the mid-thigh toward the base of the skull. Body parts below the ischia and above the eyes are estimated by using extrapolation factors for two tissues of interest (k_muscle_ for muscles and k_fat_ for fat) calculated on the CT atlases as the mean ratio of whole-body voxels of fat (or muscle) divided by the numbers of voxels of fat (or muscle) between the ischia to the eyes. This segmentation generates three types of mask from ischia to eyes: the first for the body shape, the second for the abdominal cavity and the third for the muscles. From the three masks are extracted the fat voxels, the visceral fat voxels and the muscles voxels from ischia to eyes by using Hounsfield Unit (HU) thresholds of -190 to -30 for fat voxels and -29 to + 150 for muscle voxels. Then, MBM, FBM, LBM, VFM and SCFM are calculated as shown in Fig. 1.

### Statistical analysis

Characteristics of the sample were described using counts and percentages for qualitative variables, and median and extreme values for quantitative variables. Comparison of sample characteristics according to sarcopenia was established by a Chi-squared test (or Fisher's exact test) for qualitative variables, and a Student's (or nonparametric Wilcoxon Mann Whitney) test for quantitative variables. A risk alpha level of 5% was retained for each test.

Overall and progression-free survival curves were estimated by the Kaplan–Meier method. The Log-rank test and a Cox model were used to compare overall and progression-free survival based on patients' skeletal muscle mass status, clinical, and nutritional characteristics.

Prognostic values of anthropometric parameters on overall survival were established by Receiver Operating Characteristic (ROC). When Area Under the Curves (AUC) were significantly greater than 0.5, an optimal threshold was defined by simultaneously maximizing sensitivity and specificity. A survival analysis according to this factor, both as a continuous variable (Cox model) and as a binary variable dichotomized by the threshold (log-rank test) thus defined, was then carried out to confirm the prognostic value.

Significant variables in univariate analysis (p < 0.05) were then included in a multivariate survival analysis model. Note that, to be restrictive in the variable selection due to small sample size, anthropometric parameters will be selected according to Cox model p-value < 0.05 by considering variable as continuous. Then, a model selection was carried out using a stepwise approach to successively include and remove parameters in order to obtain a unique model containing only the most relevant variables.

### Ethics

The study protocol has been approved by the Centre Henri Becquerel Cancer scientific and ethic committee (IRB 2104B). The informed consent was lifted by the Centre Henri Becquerel Cancer scientific and ethic committee. All the date were anonymized. All methods were carried out in accordance with the relevant guidelines and regulations.

### Consent for publication

All the authors give consent for publication.

## Results

**Figure 2:**
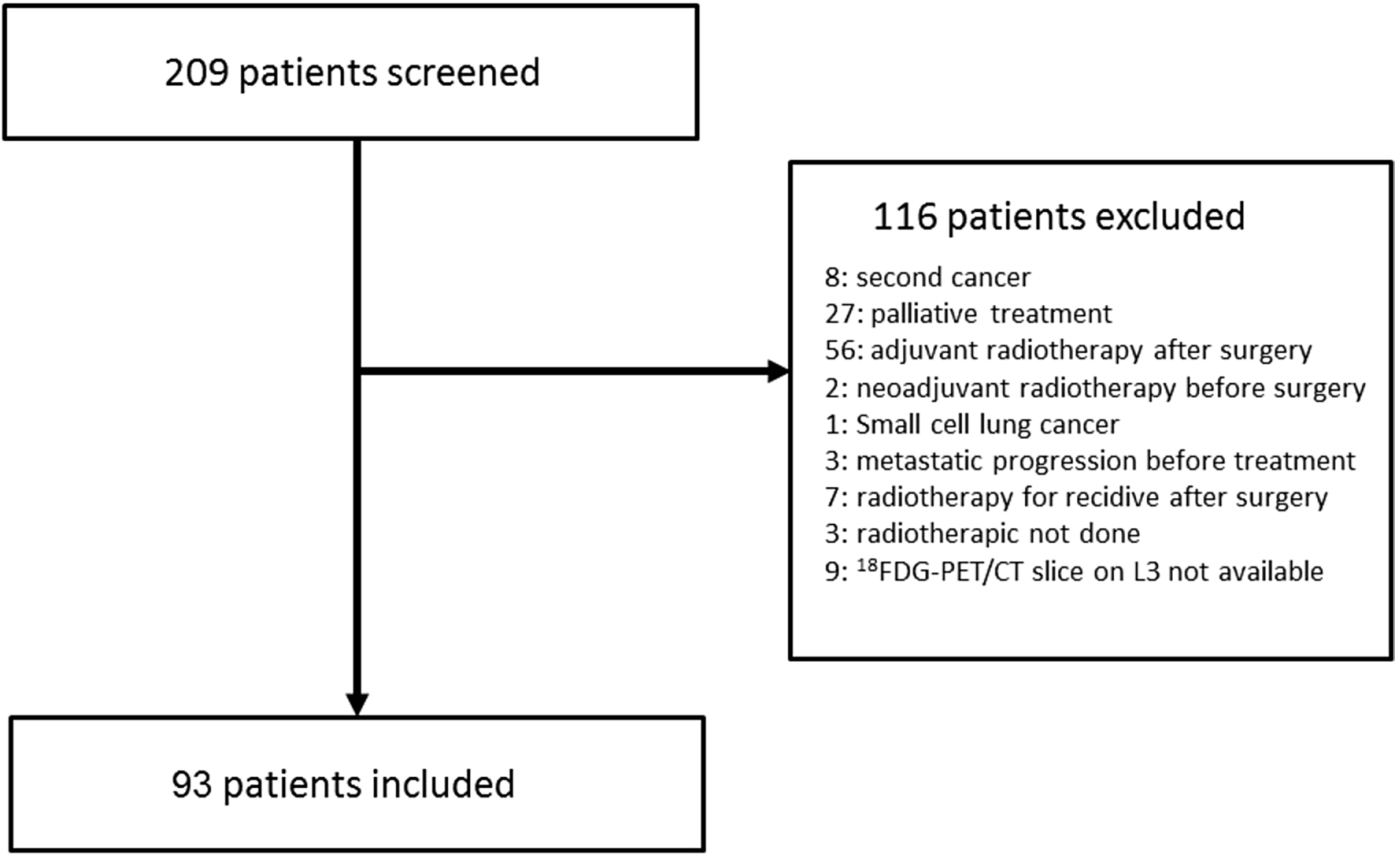
Flowchart of the study.

Between 2012 and 2015, 209 patients were addressed for radiotherapy of a lung cancer to the Centre Henri Becquerel. Flow-chart is shown in Fig. 2. The final sample included 93 patients.

The general and nutritional characteristics of the sample are described in Table 1. Based on French recommendations for diagnosis of malnutrition^15^, only 4 (4.1%) patients had a BMI below standard for age, 13 (13.9%) using albumin serum levels (13.9%), and 28 (30.1%) using weigh loss histories. The majority of patients (77/93, 72.8%) was addressed for a stage III NSCLC treatment. Patients of stage I and II were addressed because of surgical contraindications and were treated with conventional radiotherapy, because pulmonary stereotactic radiotherapy was not available in our centre at the time of treatment. Adenocarcinoma was assessed in 50% of women and 37% of men.

**Table 1:** General and nutritional characteristics of the sample, low skeletal muscle mass and non-low skeletal muscle mass patients.

	Number of patients (n = 93)	Low skeletal muscle mass (n = 41)	Non-low skeletal muscle mass (n = 52)	p
**Clinical parameters**				
Age	63 (28–87)	68 (38–87)	61 (28–85)	0.016
**Sex**				0.36
Men	73 (78.5%)	34 (82.9%)	39 (75%)	
Women	20 (21.5%)	7 (17.1%)	13 (25%)	
**Histology**				0.66
Adenocarcinoma	37 (39.8%)	14 (34.1%)	23 (44.2%)	
Squamous cell carcinoma	40 (43%)	18 (43.9%)	22 (42.3%)	
Other histology (except SCLC)	16 (17.2%)	9 (22%)	7 (13.5%)	
**TNM stage**				0.25
I	4 (4.3%)	3 (7.3%)	1 (1.9%)	
II	12 (13%)	6 (14.7%)	6 (11.5%)	
IIIA	20 (21.5%)	7 (17.1%)	13 (25%)	
IIIB	57 (61.3%)	25 (61%)	32 (61.5%)	
**Tumour localisation**				
Right lung	54 (58.1%)	6 (14.1%)	9 (17.3%)	
Left lung	30 (32.2%)	18 (43.9%)	11 (21.2%)	
Hilar	7 (7.5%)	5 (12.2%)	3 (5.8%)	
Multifocal	1 (1.1%)	1 (2.4%)	0 (0%)	
Mediastinal	1 (1.1%)	0 (0%)	1 (1.9%)	
**WHO score**				0.16
0	47 (50.5%)	17 (41.5%)	30 (57.7%)	
> = 1	46 (49.5%)	24 (58.5%)	22 (42.3%)	
SUV max*	13.99 (3.8–41.2)	13.6 (3.8–41.2)	14 (6–26.6)	0.039
PTV (cm^3^)	440.4 (85–1363.9)	440.4 (85–1363.9)	434.1 (104.3–1087.4)	0.94
**Nutritional parameters**				
Weight (kg)*	70 (37–107)	65 (37–85)	75 (45–107)	< 0.001
Usual weight (kg)*	74 (40–115)	69.5 (40–93)	76 (45–115)	0.013
Weight loss (%)*	− 1.41 (+ 11.11 to − 23.1)*	− 4.45 (+ 4.08 to − 23.1)	0 (1.56 to − 5.6)	0.008
BMI (kg/m^2^)*	23.81 (15.2–37.8)	22 (3.2)	26.1 (4.2)	< 0.001
Serum albumin level (g/l)*	37.25 (18.8–49.1)	35.3 (22–46.9)	39 (18.8–49.1)	0.01
NRI*	96.86 (65.77–116.28)	93.9 (71.4–112.9)	100 (65.8–116.3)	0.03

*Quantitative parameters are described by their median and minimal/maximum values.

Based on thresholds defined by Prado (52.4 cm/^2^m^2^ for men and 38.4cm^2^/m^2^ for women), 41 patients (44.1%) had low skeletal muscle mass. General and nutritional parameters of low skeletal muscle mass and non-low skeletal muscle mass patients are described in Table 1. Low skeletal muscle mass patients were older, had a smaller weight, a bigger weight loss history, an inferior albumin serum level and consequently inferior BMI and NRI. Non low skeletal muscle mass patients were more often treated by exclusive radiotherapy, but there was no difference for the choice of chemotherapy regimen, the dose and duration of radiotherapy.

Anthropometric parameters are described in Table 2. The Pearson correlation coefficient for SMI and Muscular Mass was 0.88 [0.83–0.92] (p < 0.001), showing an excellent correlation for the measure of the muscular mass by these two methods.

**Table 2:** Anthropometric parameter for low skeletal muscle mass and non-low skeletal muscle mass patients.

Anthropometric parameters	Low skeletal muscle mass (n = 41)	Non-low skeletal muscle mass (n = 52)	p
**Semi-automatic segmentation**			
Skeletal muscular mass L3 (SML3) (cm^2^)*	132.2 (75–156.9)	169 (103.5–246.4)	< 0.0001
Visceral fat mass L3 (VFML3) (cm^2^)*	116.6 (1.6–422.7)	154.34 (4.3–513.8)	0.13
Subcutaneous fat mass L3 (SCFML3) (cm^2^)*	107.3 (3.7–206.7)	137.1 (10.4–318)	0.001
Skeletal muscular area (SMA) (cm^2^/m^2^)*	44.9 (31.6–52.1)	56.52 (39.4–77.7)	< 0.0001
**Anthropometer3D**			
Fat body mass (FBM) (kg/m^2^)*	5.9 (0.8–11.6)	7.3 (1.3–16.3)	0.015
Visceral fat mass (VFM) (kg/m^2^)*	1.1 (0.1–3)	1.4 (0.7–1.9)	0.09
Subcutaneous fat mass (SCFM) (kg/m^2^)*	4.7 (0.7–8.8)	5.7 (1.1–12.7)	0.011
Lean body mass (LBM) (kg/m^2^)*	16.6 (11.5–20.7)	19.4 (13.6–22.1)	< 0.0001
Muscle body mass (MBM) (kg/m^2^)*	8.1 (5.5–10.4)	10 (5.9–13)	< 0.0001

*Quantitative parameters are described by their median and minimal/maximum values.

**Figure 3:**
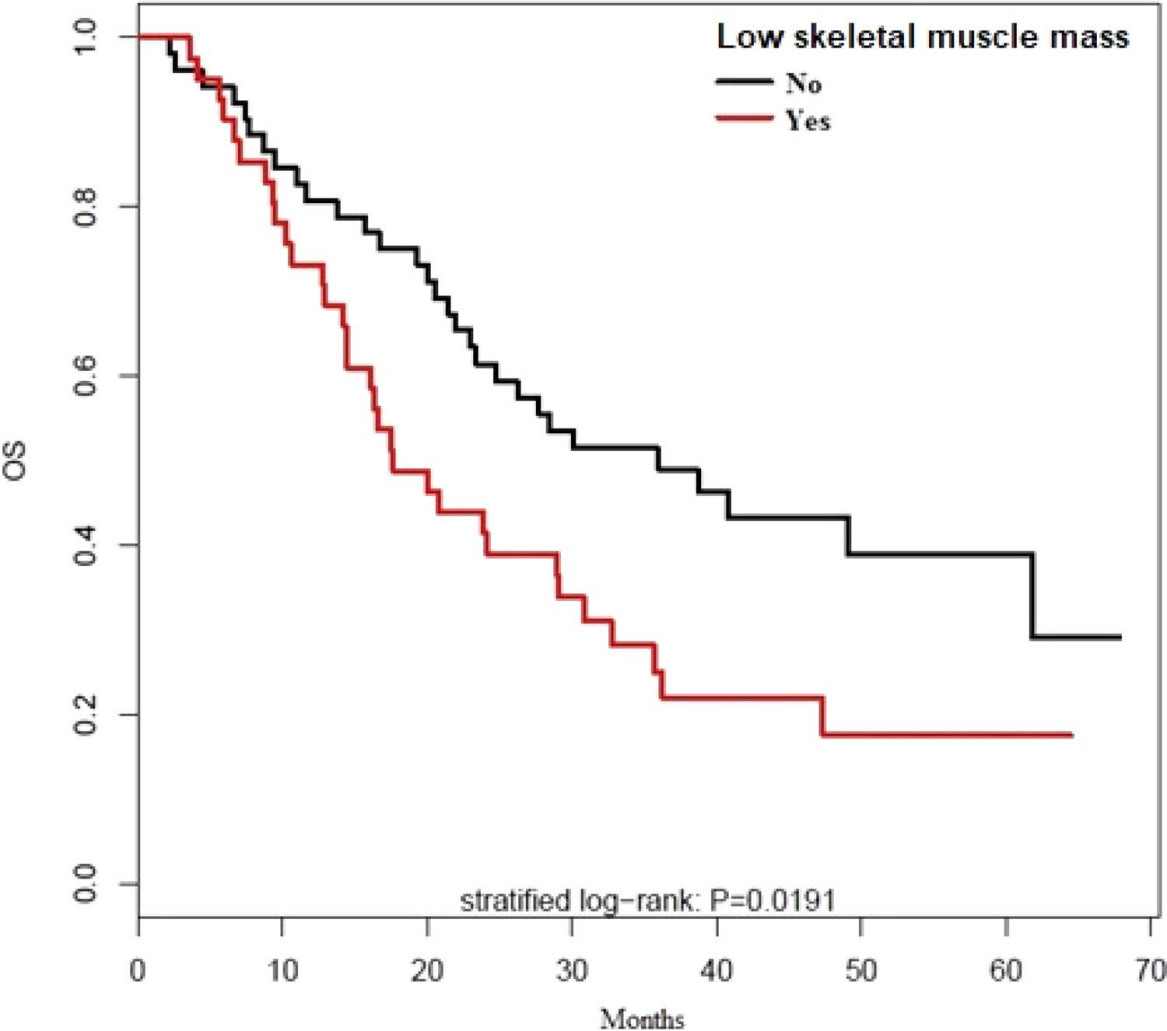
Overall survival for low skeletal muscle mass.

At date of analysis, 62 (66.7%) patients were dead, whose 32 of the 41 low skeletal muscle mass patients (78%) and 30 of the non-low skeletal muscle mass patients (57%). The median overall survival was 26.2 months, 17.6 months for low skeletal muscle mass patients versus 36 months for non-low skeletal muscle mass patients (p = 0.02, see Fig. 3).

**Figure 4:**
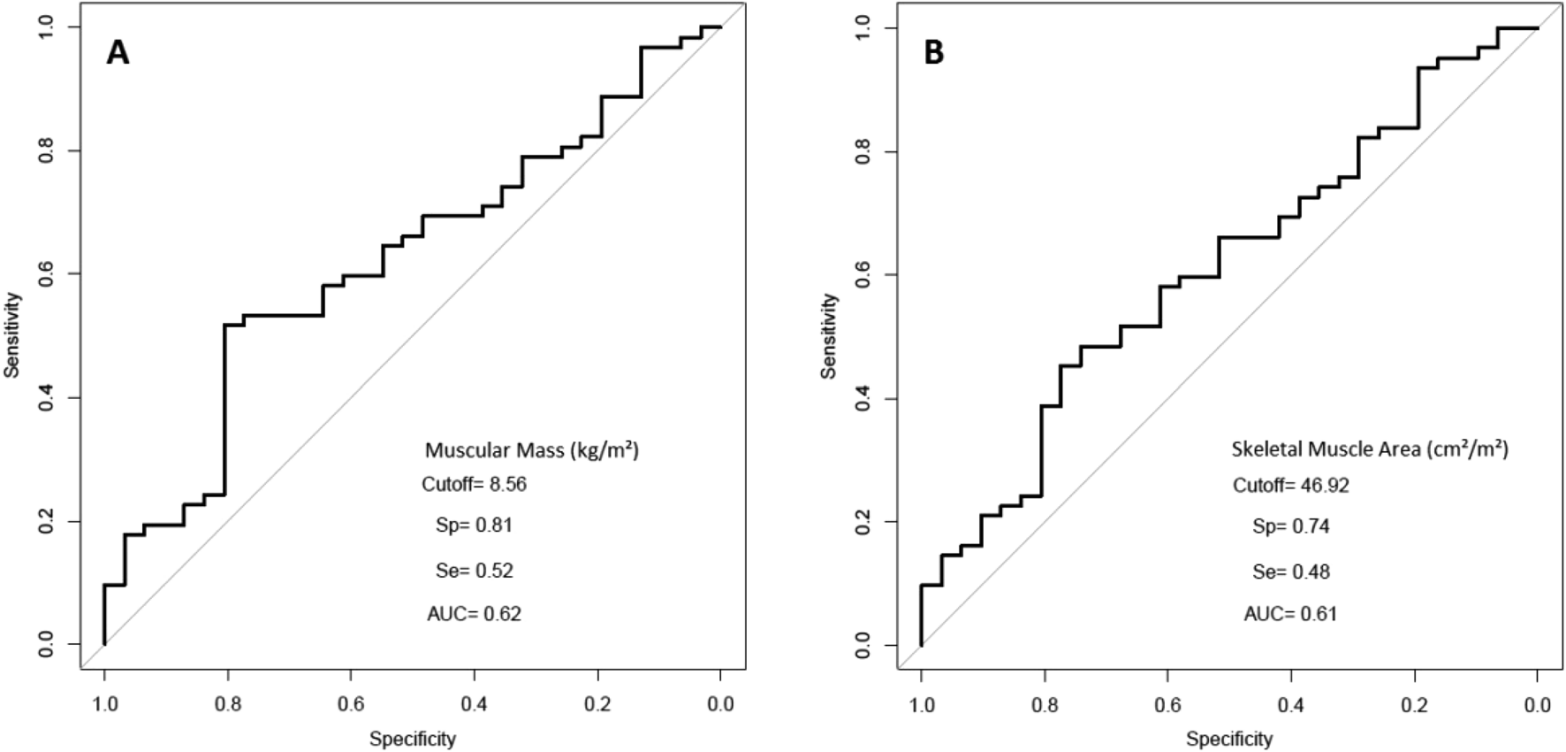
ROC curve and AUC for muscular mass (**A**) and skeletal muscle area (**B**).

In exploratory analysis, in the determination of predictive anthropometric factors on survival by means of ROC curves (Table 3), optimal thresholds have been defined for Muscular Mass (kg/m^2^) and Skeletal Muscular Index (cm^2^/m^2^) (Fig. 4a,b). With a threshold of 8.56 kg/m^2^ for Muscular Mass (AUC = 0.62, p = 0.03), an overall survival median time of 16.1 months was observed for the 37 patients below this threshold, and of 38.7 months for the others (HR = 2.35 [1.42–3.89], p = 0.021). With a threshold of 46.92 cm^2^/m^2^ for Skeletal Muscular Index (AUC = 0.61, p = 0.04), an overall survival median time of 17.1 months was observed for the 38 patients below this threshold, and of 35.7 months for the others (HR = 1.92 [1.16;3.17], p = 0.011). In univariate survival analysis, BMI < 21, albumin serum level < 35 g/l and NRI < 97.5 were associated with worse overall survival while WHO score = 0 was associated with a better overall survival. As concerning anthropometric parameters, SMI < 46.92 cm^2^/m^2^, low skeletal muscle mass (defined by Prado and Muscular < 8.56 kg/m^2^) were associated with a worse overall survival (Table 4). Due to numerous missing value of NRI and Serum albumin levels (respectively 35 and 33), these variables has not been included in the multivariable model. In multivariate analysis, the only factors associated with overall survival were WHO score = 0 and Muscular Mass < 8.56 kg/m^2^ (Table 4). Sex cut-offs values has been computed for each parameter, displayed in Supplementary table 1.

**Table 3:** ROC thresholds values and AUC.

Parameter	Threshold	AUC	Sensitivity	Specificity
Fat body mass (kg/m^2^)	4.16	0.55	0.34	0.9
Visceral fat mass (kg/m^2^)	1.25	0.57	0.55	0.55
Subcutaneous fat mass (kg/m^2^)	3.52	0.55	0.34	0.9
Lean body mass (kg/m^2^)	17.31	0.56	0.48	0.71
Muscle body mass (kg/m^2^)	8.56	0.62	0.52	0.81
Skeletal muscular mass L3 (cm^2^)	159.9	0.57	0.71	0.45
Visceral fat mass L3 (VFML3)	48.88	0.56	0.58	0.55
Subcutaneous fat mass L3 (SCFML3)	37.44	0.55	0.44	0.87
Skeletal muscular area (SMA)	46.92	0.6	0.48	0.74

**Table 4:** Univariate analysis and Final result of multivariate stepwise analysis for overall survival.

Univariate analysis	Hazard ratios			P (cox continuous)
	Value (95% C.I.)	Reference	p	
Fat Body mass (FBM)	2.2 (1.3–3.7)	≥ 4.16 kg/m^2^	< 0.01	0.1
Visceral fat mass (VFM)	1.3 (0.8–2.2)	≥ 1.25 kg/m^2^	0.3	0.13
Subcutaneous Fat Mass (SCFM)	2.2 (1.3–3.7)	≥ 3.52 kg/m^2^	< 0.01	0.11
Lean body mass (LBM)	1.7 (1–2.7)	≥ 17.31 kg/m^2^	0.05	0.29
Muscle body mass (MBM)	2.4 (1.4–3.9)	≥ 8.56 kg/m^2^	** < 0.001**	**0.021**
Skeletal muscular mass L3 (SML3)	1.6 (0.9–2.7)	≥ 159.9 cm^2^	0.11	0.15
Visceral fat mass L3 (VFML3)	1.4 (0.9–2.3)	≥ 48.88 cm^2^	0.17	0.175
Subcutaneous fat mass L3 (SCFML3)	2.6 (1.5–4.2)	≥ 37.44 cm^2^	< 0.001	0.28
Skeletal muscular area (SMA)	1.9 (1.1–3.1)	≥ 46.92cm^2^/m^2^	**0.01**	**0.033**
Sarcopenia*	1.8 (1.1–3)	No	**0.02**	–
BMI**	1.8 (1–3.1)	≥ 21	**0.05**	–
Serum albumin level ** (33 missing values)	2.2 (1.2–4.1)	≥ 35 g/L	0.01	–
NRI*** (35 missing values)	2.3 (1.2–4.3)	≥ 97.5	0.01	–
WHO score	0.5 (0.3–0.9)	> 0	**0.01**	–
Age	1.18 (0.71–1.95)	≤ 63	0.52	**0.068**
Multivariate stepwise analysis	Value (95% C.I.)(95%CI)	Reference	p	
WHO score	0.6 (0.4–0.9)	> 0	**0.042**	
Muscle body mass (MBM)	2.1 (1.3–2.6)	≥ 8.56 kg/m^2^	**0.003**	

*Defined by Prado; ** Threshold based on French recommendations for prevention of the undernutrition; ***Threshold based on Buzby.

**Figure 5:**
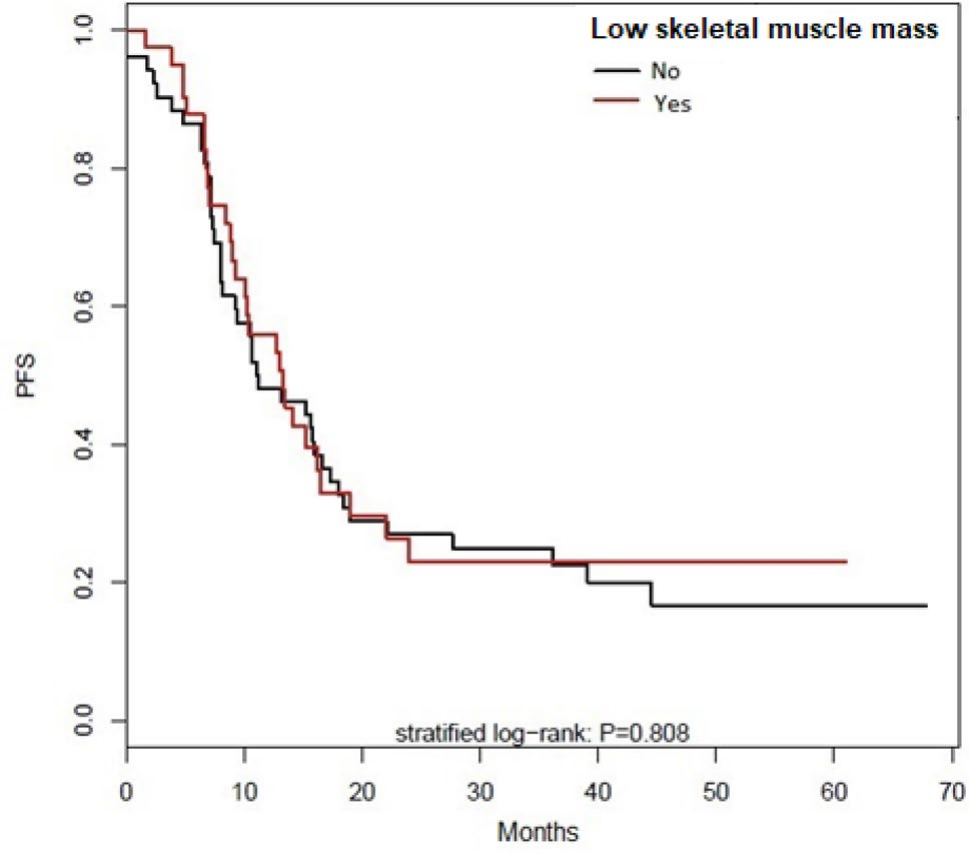
Progression free survival for low skeletal muscle mass.

At date of analysis, 70 patients had progressed or relapsed, whose 28 of the 41 low skeletal muscle mass patients (68%) and 42 of the non-low skeletal muscle mass patients (80.8%). The median progression free survival was 12.9 months, 13.3 months for low skeletal muscle mass patients and 11.2 months for non-low skeletal muscle mass patients (p = 0.81, see Fig. 5). None of studied parameters were shown to be statistically associated by progression free survival in univariate analysis.

During treatment, 59 patients (63%) presented grade ≥ 3 toxicities. Thirty-eight patients (49%) needed a chemotherapy dose reduction and 43 (55%) needed chemotherapy report more than 15 days. Fifteen patients (16%) had an interruption of radiotherapy for more than 3 days, and 86 patients (92.4%) received the radiotherapy dose prescribed. There was only a significant difference according to low skeletal muscle mass for cardiac toxicities, with 12.9% of low skeletal muscle mass patients and 5.1% of non-low skeletal muscle mass patients (p = 0.02). The mainly observed toxicity was neutropenia, but rarely induced febrile neutropenia, precisely only 4 patients (12.5%), all were low skeletal muscle mass. Toxicities were not associated with anthropometric parameters measured by Anthropometer3D.

## Discussion

The impact of low skeletal muscle mass has been increasingly studied over the last 10 years and many studies confirm its prognostic interest. However, data in patients treated with radiotherapy or radiochemotherapy remain limited.

For NSCLC treated by RTCT, there is to our knowledge only one study, but which only included 41 patients, the lack of power of the study did not allow to highlight the prognostic character of low skeletal muscle mass (25 minus versus 53 months p = 0.13)^16^. Il a study of stage I lung cancer treated by stereotactic radiotherapy, skeletal muscle mass had an impact on survival^17^.

So our best knowledge, our study is the first to show a pronostic impact of low skeletal muscle mass for patients treated by exclusive radiochemotherapy for a NSCLC. These results are consistent with those described by Prado and Martin^10,18^, show that low skeletal muscle mass (considered as a reflect of sarcopenia) is a more powerful prognostic factor than usual nutritional parameters like weight lost and serum albumin levels and confirm the importance to evaluate low skeletal muscle mass before starting treatments. The SMI was not predictive of overall survival in our study, but a muscular mass < 8.56 kg/m^2^ was. However, there was a strong correlation coefficient between these two measures. Decazes has shown that these measurements were correlated (and that muscle mass measured by Anthropometer 3 D can be used instead of SMI^24^. Both systems had equivalent survival prediction but different sensitivities and specificities secondary to different cutt off.

A review confirms the interest of low skeletal muscle mass specifically in lung cancer but the included studies do not specifically study the stage III population treated with radiochemotherapy^19^. The high prevalence of low skeletal muscle mass in this population, confirmed by our study (44%), calls for optimised management even if low skeletal muscle mass was not associated with increased toxicity. However, this may be secondary to the lack of power and the small number of patients who had to have their treatment interrupted. In our study, the CT slice comes from ^18^FDG-PET/CT examination, with acquisition parameters different from those used by Prado, like the slice thickness: we use a 2 mm slice thickness, while Martin used a 5 mm slice thickness and Prado didn’t described the acquisition parameters. Fuch and al showed that 5 mm slice thickness significantly increased muscle sectional area by 1.11% compared to 2 mm (p < 0.001)^20^. It could lead to a bad classification of patients by underestimate muscular area at L3 in our study. But we also showed that low skeletal muscle mass can be evaluated by automatic segmentation on every slice of the ^18^FDG-PET/CT examination and it should reduce the impact of slice thickness.

Mechanisms by which low skeletal muscle mass confers increased mortality have not been actually explained. Several theories can be suggested to explain the effect: an increase of susceptibility to nosocomial infection^21^, baseline systemic inflammation associated with a more frequent rate of metastasis and progression^22,23^, and an impact of low skeletal muscle mass on chemotherapy volume and distribution^7,8^. The absences of significant differences for progression free survival and toxicities in our study suggests that low skeletal muscle mass is probably more a powerful reflect of the patient’s general condition than the tumour aggressiveness.

Some limitations of our study should be noted, especially as concerning the sample size and few events observed. Indeed, at date of analysis, 62 patients were dead, whose 32 of the 41 patients with low skeletal muscle mass and 30 of the other patients. Moreover, as low skeletal muscle mass was observed in only 41 participants, yet the majority of analyses are based on this very small group, thus limiting interpretation of our results. Then, sarcopenia is defined by Prado et al. by using sex specific cut-offs. Due to the small number of women in our sample (n = 20), predictive performances of these female specific cut-offs need to be considered.

To conclude, low skeletal muscle mass assessed by CT sequence on ^18^FDG-PET/CT at baseline is an independent and robust prognostic factor for overall survival in patients with NSCLC treated by exclusive radiochemotherapy. These easily gathered imaging features can identify an at-risk population who need a specific therapy. The use of Anthropometer3D can provide a current evaluation of skeletal muscle mass at practice.

## Supplementary Information

Supplementary Information.

## Data Availability

All the data are available at Centre Henri Becquerel, ROUEN. Please contact the corresponding author if interested.

## Abbreviations

BMI: Body mass index
SMA: Skeletal muscle area
NSCLC: Non small cell lung cancer
^18^FDG-PET/CT: Fluorodeoxyglucose-positron emission tomography/computerized tomography
WHO: World health organization
NRI: Nutritional risk index
CRT: Chemo-radiotherapy
CTCAE: Common terminology criteria for adverse events
SUV_Max_: Standardized uptake value maximum
PACS: Picture archiving and communication system
SMI: Skeletal muscle index
VFM: Visceral fat mass
SCFM: Subcutaneous fat mass
LBM: Lean body mass
FBM: Fat body mass
MBM: Muscle body mass
VFM: Visceral fat mass
SCFM: Subcutaneous fat mass
HU: Hounsfield unit
ROC: Receiver operating characteristic
AUC: Area under the curves
