# Supplementary material for: Prognostic value of low skeletal muscle mass in patient treated by exclusive curative radiochemotherapy for a NSCLC
Source: Sci Rep. 2021 May 20;11:10628. doi: 10.1038/s41598-021-90187-6 (PMC8137692; doi:10.1038/s41598-021-90187-6)
Supplement: Supplementary file 1 — Supplementary Information. [file 41598_2021_90187_MOESM1_ESM.docx]

Supplementary table 1: ROC thresholds values and AUC

|  | **TOTAL (n=93)** | | | | **MEN (n=73)** | | | | **WOMEN (n=20)** | | | |
| --- | --- | --- | --- | --- | --- | --- | --- | --- | --- | --- | --- | --- |
| **Parameter** | **Thresh-old** | **AUC** | **Se** | **Sp** | **Thresh-old** | **AUC** | **Se** | **Sp** | **Thresh-old** | **AUC** | **Se** | **Sp** |
| **Fat Body Mass (kg/m²)** | 4.16 | 0.55 | 0.34 | 0.9 | 4.16 | 0.51 | 0.31 | 0.92 | 3.75 | 0.68 | 0.46 | 0.86 |
| **Visceral Fat Mass (kg/m²)** | 1.25 | 0.57 | 0.55 | 0.55 | 1.25 | 0.56 | 0.45 | 0.67 | 0.58 | 0.6 | 0.62 | 0.57 |
| **Subcutaneous Fat Mass (kg/m²)** | 3.52 | 0.55 | 0.34 | 0.9 | 3.52 | 0.5 | 0.31 | 0.92 | 4.72 | 0.7 | 0.54 | 0.86 |
| **Lean Body Mass (kg/m²)** | 17.31 | 0.56 | 0.48 | 0.71 | 18.93 | 0.54 | 0.61 | 0.54 | 16.82 | 0.69 | 0.85 | 0.57 |
| **Muscle Body Mass (kg/m²)** | 8.56 | 0.62 | 0.52 | 0.81 | 8.67 | 0.63 | 0.43 | 0.88 | 6.71 | 0.73 | 0.46 | 1 |
| **Skeletal Muscular Mass L3 (cm²)** | 159.9 | 0.57 | 0.71 | 0.45 | 159.9 | 0.58 | 0.63 | 0.58 | 108.3 | 0.73 | 0.69 | 0.71 |
| **Visceral Fat Mass L3 (VFML3)** | 48.88 | 0.56 | 0.58 | 0.55 | 48.88 | 0.56 | 0.51 | 0.67 | 11.32 | 0.57 | 0.54 | 0.71 |
| **Subcutaneous Fat Mass L3 (SCFML3)** | 37.44 | 0.55 | 0.44 | 0.87 | 37.44 | 0.49 | 0.41 | 0.88 | 39.95 | 0.71 | 0.62 | 0.86 |
| **Skeletal Muscular Area (SMA)** | 46.92 | 0.61 | 0.48 | 0.74 | 52.9 | 0.63 | 0.57 | 0.67 | 41.77 | 0.68 | 0.62 | 0.71 |
